# Focussing on the origins of preterm birth: Why understanding aetiology is critical to optimising outcomes

**DOI:** 10.1371/journal.pmed.1004601

**Published:** 2025-05-20

**Authors:** Jennifer Jardine, Laura Goodfellow, Caroline Ovadia, Anna L. David, Catherine Williamson

**Affiliations:** 1 Department of Obstetrics and Gynaecology, University of Cambridge, Cambridge, United Kingdom; 2 Centre for Public Health and Policy, Wolfson Institute of Population Health, Queen Mary University of London, London, United Kingdom; 3 Department of Women’s and Children’s Health, University of Liverpool, Liverpool, United Kingdom; 4 Department of Women and Children’s Health, King’s College London, London, United Kingdom; 5 Elizabeth Garrett Anderson Institute for Women’s Health, University College London, London, United Kingdom; 6 Department of Metabolism, Digestion and Reproduction, Faculty of Medicine, Imperial College London, London, United Kingdom

## Abstract

Preterm birth is a central determinant of infant morbidity and mortality. Efforts to reduce its incidence must address the disparate underlying causes.

Between 6% and 13% of all babies are born before term, defined as before 37 weeks’ gestation [1]. Preterm birth is the primary underlying contributor to childhood morbidity and mortality, with the impact highest in lower-resource settings and low- and middle-income countries (LMICs) [1]. There is, therefore, a substantial imperative to lower rates worldwide. In the UK, the Government has set a target to reduce overall rates from 8% to 6%, established national initiatives to decrease preterm birth in at-risk women, and issued recommendations from a recent Inquiry aimed at decreasing the incidence and impact of preterm birth [2].

However, targeting preterm birth as a single outcome has substantial limitations: as crude a concept as adult death before 37 years [3], and this blunt grouping risks conflating multiple pathways and solutions. In this Perspective, we highlight the different aetiologies of preterm birth and explain how clarification of underlying pathways is critical to creating appropriate targets, research priorities, and individualised prophylactic and therapeutic interventions.

## Classification and aetiologies

Preterm birth can be broadly split into three categories: (1) spontaneous, where contractions coupled with cervical dilatation are the antecedent event; (2) birth preceded by preterm, prelabour rupture of fetal membranes (PPROM); and (3) iatrogenic, initiated by healthcare practitioners to benefit maternal and/or fetal health [4]. The proportion of preterm babies born in each category varies substantially, with recent estimates in high-income countries being 30%–78% spontaneous, 10%–30% PPROM and 22%–55% iatrogenic [5].

**Fig 1:**
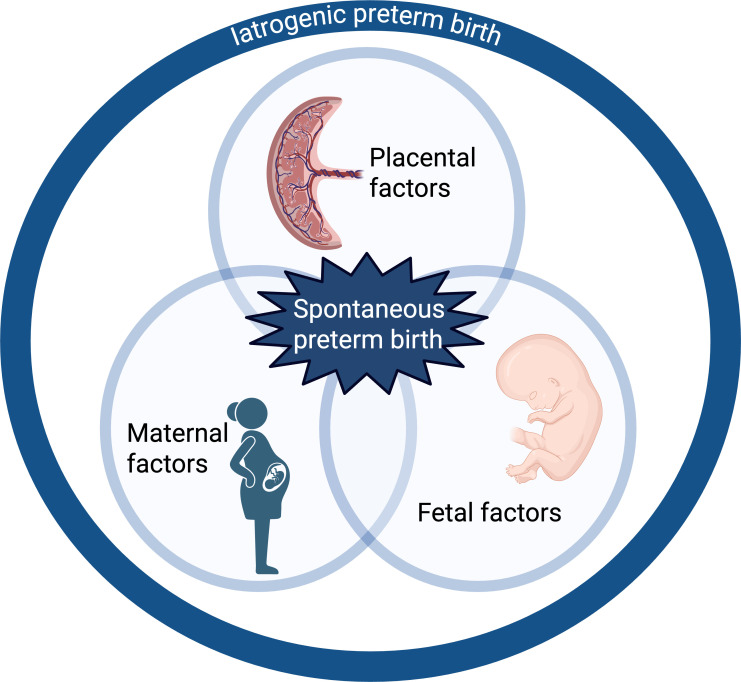
“Triple Risk” model for preterm birth. Created in BioRender. Jardine, J. (2025) https://BioRender.com/r42v086.

This separation into “how it started” itself masks overlapping underlying pathologies, including placental insufficiency and fetal membrane ageing; uterocervical integrity; interactions between the maternal immune system, microbiota, and infection; metabolic health; and other fetal and maternal conditions that may limit gestation [3,4]. For each, ideal preventive strategies are likely to differ, and multiple different pathologies may contribute to an individual preterm birth event. Furthermore, the distribution of these aetiologies differs between singleton and multifetal pregnancies, of which half end before 37 weeks. When calculating preterm birth rates, the inclusion of multifetal pregnancies, or births (if infants are the population analysed) can substantially change overall findings, as multifetal pregnancies are disproportionately over-represented in the preterm population. In both observational and interventional studies regarding preterm birth, it is therefore important to specify the population analysed, and the types and mechanisms of preterm birth being considered. We propose a “triple risk” model, akin to that of stillbirth [6], to describe the various contributors to preterm birth (Fig 1).

## Untangling mechanisms

Current targets to reduce preterm birth largely focus on overall rates. This fails to consider underlying differences in drivers of preterm birth. Preterm birth can either be provider-initiated, where preterm birth is a clinical choice made with the aim to achieve optimum maternal and fetal/neonatal outcomes given underlying pathology; or spontaneous, where birth begins and cannot be stopped. Data from high-income settings suggest that rates of iatrogenic preterm births have increased and spontaneous preterm births have fallen [4,5]. If we do not account for these distinct categories separately, we neither see improvement for specific subgroups of cases—leading potentially to a lack of faith in interventions made—nor see changing opportunities for intervention. Yet, multiple classification systems of preterm birth exist, with differing emphasis upon whether the birth was spontaneous or iatrogenic, and, within those, the underlying mechanisms [3,7,8]. Despite their existence, such systems are little used, including in research studies, and as a result there are limited mentions of classifications or targeted interventions in guideline documents for prevention of preterm birth [9].

## Benefits of rigorous use of classification

More rigorous use of classification systems would enable clarity about choice of intervention. Available interventions include optimising pre-pregnancy maternal health, addressing smoking status, aspirin to prevent pre-eclampsia, surveillance for urinary tract infection, identification and management of gestational disease, vaginal progesterone, cervical cerclage, and measures to improve neonatal outcome when preterm birth seems likely. These interventions are best delivered by different specialists, and some, particularly cerclage, carry risks that require justification with a clear indication.[10]

Furthermore, research on more homogeneous study groups improves the chance of identifying successful interventions. When different aetiologies of preterm birth are combined in the same study group, it risks diluting the benefit of predictive biomarkers or interventions. In addition to delaying clinical adoption, equivocal results induce disillusionment towards preterm birth care and reduce success with funding applications, hindering ongoing research.

In policy, recommendations should focus on funding and research that will drive a clearer understanding of the distinct pathological mechanisms underlying preterm birth, and thus inform targeted intervention. The urgent need to determine which interventions would most effectively support preterm birth prevention in each woman was highlighted in the recent UK Parliamentary inquiry [2].

Finally, some causes of preterm birth have implications for women’s lifelong health; gestational conditions that lead to preterm birth are independent risk factors for maternal illness such as diabetes, cardiovascular conditions, and premature mortality [11]. Better understanding of the underlying aetiologies could allow targeted intervention to improve the long-term health of both mothers and babies.

## Challenges of classification

Applying aetiological classification systems to preterm births is complicated by the interplay of multiple underlying pathological drivers [3,4] Clinicians must identify which component pathologies are present, and how they interact to contribute to each birth. This can be hampered by lack of resources: in LMICs, insufficient access to ultrasound scans to accurately date the pregnancy can make it difficult to determine the gestational age at birth, and limited healthcare access can result in a failure to detect or record co-morbidities, such as gestational diabetes or hypertension.

Clinical presentation of preterm birth overlaps with that of late miscarriage, raising questions about the earliest gestation of birth that should be included in its definition. Improved electronic health record data may accelerate more granular reporting and could enhance insights into subsequent maternal and child health with linkage to additional health and education records [5].

## Future developments

Recent initiatives, such as FIGO PremPrep-5, a bundle of five interventions to reduce morbidity for the baby born preterm, aim to improve neonatal outcomes when a preterm birth is inevitable [12]. Future developments to better understand preterm birth include enhanced research into underlying genetic risks and pathological mechanisms, identification of at-risk populations, investigating the impact of adverse societal and economic influences including climate change and structural racism, and evaluation of targeted interventions. This evaluation should include consideration of the population-attributable risk, which estimates the burden of disease caused by a risk factor. Implementing interventions late in pregnancy, such as progesterone and cerclage, may only make a very small reduction to the preterm birth rate at a population level, whereas earlier interventions, such as improving family planning, preconception health, and HPV vaccination to avoid CIN treatment, may have more widespread impact.

## Conclusions

The term ‘preterm birth’ masks a complex and heterogenous set of overlapping phenotypes, reflecting a broad range of underlying aetiologies. Without an attempt to classify preterm births at a more granular level, we risk obstructing the identification of interventions, research studies, and policies that have the potential to improve outcomes for women and babies. We strongly recommend focussing on the aetiology of preterm birth and not purely the outcome, to drive reductions in its prevalence and impact.

## Abbreviation

LMICs: low- and middle-income countries
PPROM: preterm, prelabour rupture of fetal membranes

## References

[1] Ohuma EO, Moller A-B, Bradley E, Chakwera S, Hussain-Alkhateeb L, Lewin A, et al. National, regional, and global estimates of preterm birth in 2020, with trends from 2010: a systematic analysis. Lancet. 2023;402(10409):1261–71. doi: 10.1016/S0140-6736(23)00878-4 37805217

[2] Committee House of Lords Preterm Birth. Preterm birth: reducing risks and improving lives [Internet]. 2024 Nov. Available from: https://publications.parliament.uk/pa/ld5901/ldselect/ldpreterm/30/30.pdf

[3] Villar J, Cavoretto PI, Barros FC, Romero R, Papageorghiou AT, Kennedy SH. Etiologically based functional taxonomy of the preterm birth syndrome. Clin Perinatol. 2024;51(2):475–95. doi: 10.1016/j.clp.2024.02.014 38705653 PMC11632914

[4] Goldenberg RL, Culhane JF, Iams JD, Romero R. Epidemiology and causes of preterm birth. Lancet. 2008;371(9606):75–84. doi: 10.1016/S0140-6736(08)60074-4 18177778 PMC7134569

[5] Aughey H, Jardine J, Knight H, Gurol-Urganci I, Walker K, Harris T, et al. Iatrogenic and spontaneous preterm birth in England: a population-based cohort study. BJOG. 2023;130(1):33–41. doi: 10.1111/1471-0528.17291 36073305 PMC10092353

[6] Warland J, Mitchell EA. A triple risk model for unexplained late stillbirth. BMC Pregnancy Childbirth. 2014;14:142. doi: 10.1186/1471-2393-14-142 24731396 PMC3991879

[7] Manuck TA, Esplin MS, Biggio J, Bukowski R, Parry S, Zhang H, et al. The phenotype of spontaneous preterm birth: application of a clinical phenotyping tool. Am J Obstet Gynecol. 2015;212(4):487.e1–487.e11. doi: 10.1016/j.ajog.2015.02.010 25687564 PMC4456184

[8] Moutquin J-M. Classification and heterogeneity of preterm birth. BJOG. 2003;110(s20):30–3. doi: 10.1016/s1470-0328(03)00021-1 12763108

[9] Jacobsson B, Simpson JL, FIGO Working Group for Preterm Birth. FIGO good practice recommendations for reducing preterm birth and improving child outcomes. Int J Gynaecol Obstet. 2021;155(1):1–4. doi: 10.1002/ijgo.13863 34520060

[10] van Dijk CE, Breuking SH, Jansen S, Limpens JCEJM, Kazemier BM, Pajkrt E. Perioperative complications of a transvaginal cervical cerclage in singleton pregnancies: a systematic review and meta-analysis. Am J Obstet Gynecol. 2023;228(5):521–534.e19. doi: 10.1016/j.ajog.2022.10.026 36441090

[11] McDonald SD, Malinowski A, Zhou Q, Yusuf S, Devereaux PJ. Cardiovascular sequelae of preeclampsia/eclampsia: a systematic review and meta-analyses. Am Heart J. 2008;156(5):918–30. doi: 10.1016/j.ahj.2008.06.042 19061708

[12] Hall M, Valencia C, Soma-Pillay P, Luyt K, Jacobsson B, Shennan A, et al. Effective and simple interventions to improve outcomes for preterm infants worldwide: the FIGO PremPrep-5 initiative. Int J Gynecol Obstet. 2024;165(3):929–35. 10.1002/ijgo.15269 38264849

